# Monitoring response to neoadjuvant chemotherapy in triple negative breast cancer using circulating tumor DNA

**DOI:** 10.1186/s12885-024-12689-6

**Published:** 2024-08-16

**Authors:** Jennifer H. Chen, Sridevi Addanki, Dhruvajyoti Roy, Roland Bassett, Ekaterina Kalashnikova, Erik Spickard, Henry M. Kuerer, Salyna Meas, Vanessa N. Sarli, Anil Korkut, Jason B. White, Gaiane M. Rauch, Debu Tripathy, Banu K. Arun, Carlos H. Barcenas, Clinton Yam, Himanshu Sethi, Angel A. Rodriguez, Minetta C. Liu, Stacy L. Moulder, Anthony Lucci

**Affiliations:** 1 Department of Breast Surgical Oncology, The University of Texas MD Anderson Cancer Center, 1400 Pressler Street, FCT 7.6000, Unit 1484,, Houston, TX 77030 US; 2 Department of Biostatistics, The University of Texas M. D. Anderson Cancer Center, Houston, TX USA; 3 Natera, Inc, Austin, TX USA; 4 Department of Bioinformatics and Computational Biology, The University of Texas M.D. Anderson Cancer Center, Houston, TX USA; 5 Breast Medical Oncology, The University of Texas M. D. Anderson Cancer Center, Houston, TX USA; 6 Abdominal Imaging Department, MD Anderson Cancer Center, Houston, TX USA; 7 Breast Medical Oncology and Clinical Cancer Genetics, The University of Texas M. D. Anderson Cancer Center, Houston, TX USA; 8 Medical Oncology, Eli Lilly and Company, Indianapolis, IN USA

**Keywords:** Liquid biopsy, ctDNA, CTC, Triple negative breast cancer, Liquid biopsy in neoadjuvant setting

## Abstract

**Background:**

Triple negative breast cancer (TNBC) is an aggressive subtype with poor prognosis. We aimed to determine whether circulating tumor DNA (ctDNA) and circulating tumor cell (CTC) could predict response and long-term outcomes to neoadjuvant chemotherapy (NAC).

**Methods:**

Patients with TNBC were enrolled between 2017–2021 at The University of Texas MD Anderson Cancer Center (Houston, TX). Serial plasma samples were collected at four timepoints: pre-NAC (baseline), 12-weeks after NAC (mid-NAC), after NAC/prior to surgery (post-NAC), and one-year after surgery. ctDNA was quantified using a tumor-informed ctDNA assay (Signatera^TM^, Natera, Inc.) and CTC enumeration using CellSearch. Wilcoxon and Fisher’s exact tests were used for comparisons between groups and Kaplan–Meier analysis used for survival outcomes.

**Results:**

In total, 37 patients were enrolled. The mean age was 50 and majority of patients had invasive ductal carcinoma (34, 91.9%) with clinical T2, (25, 67.6%) node-negative disease (21, 56.8%). Baseline ctDNA was detected in 90% (27/30) of patients, of whom 70.4% (19/27) achieved ctDNA clearance by mid-NAC. ctDNA clearance at mid-NAC was significantly associated with pathologic complete response (*p* = 0.02), whereas CTC clearance was not (*p* = 0.52). There were no differences in overall survival (OS) and recurrence-free survival (RFS) with positive baseline ctDNA and CTC. However, positive ctDNA at mid-NAC was significantly associated with worse OS and RFS (*p* = 0.0002 and *p* = 0.0034, respectively).

**Conclusions:**

Early clearance of ctDNA served as a predictive and prognostic marker in TNBC. Personalized ctDNA monitoring during NAC may help predict response and guide treatment.

## Introduction

Triple negative breast cancer (TNBC) is an aggressive subtype of breast cancer that accounts for 15–20% of all cases [1]. Given the lack of targeted therapies secondary to negative hormone receptor expression and absence of human epidermal growth factor receptor 2 (HER2) amplification, current standard of care consists of neoadjuvant or adjuvant systemic therapies with definitive surgical resection and adjuvant radiotherapy if indicated [2]. Compared to non-TNBC disease, TNBC has been associated with higher rates of recurrence and worse overall and progression-free survival [3–5]. As such, there remains a need to identify both prognostic and predictive biomarkers for this patient population to better monitor disease progression and treatment response.

Liquid biopsies, including detection of circulating tumor DNA (ctDNA) and enumeration of circulating tumor cells (CTCs), offer a minimally invasive approach for disease monitoring [6–9]. Advantages of liquid biopsies over current methods of disease surveillance (routine imaging, tumor markers, tissue biopsy) include longitudinal patient-specific monitoring, convenient sample collection, and potential cost efficiency [7, 9]. More importantly, liquid biopsies might provide a means to detect molecular disease recurrence prior to radiographic evidence or clinical manifestation and predict treatment response prior to surgery. This so-called “lead-time” allows clinicians to pre-emptively intervene in a subset of patients with suboptimal response to traditional chemotherapy regimens at an earlier timepoint. In this manner, clinicians can tailor patient regimens and salvage high-risk patients or poor responders by using targeted therapies and/or enrolling patients in clinical trials testing novel therapies.

Several studies have proposed the use of ctDNA and CTCs as markers of prognosis in metastatic breast disease and as surrogates for molecular residual disease (MRD) in early-stage breast cancer [10–18]. Among patients with metastatic disease, presence of > 5 CTCs per 7.5 mL of blood has been validated as a cut-off to identify patients with significantly worse overall survival [19]. Similarly, among patients with early-stage disease, detection of ctDNA has been shown to occur with a median lead time of 8–11 months prior to clinical relapse [20, 21]. While several studies have evaluated the role of liquid biopsy to risk stratify patients for relapse, few have evaluated its use in the neoadjuvant setting [22, 23]. Given that previous studies have linked ctDNA and CTC detection in triple negative disease with recurrence and reduced survival outcomes, we sought to investigate the association of ctDNA and CTCs with pathological complete response (pCR) among patients with TNBC. Since TNBC is a high-risk subtype where up to two-thirds of patients can have residual disease at surgical resection [3] – and presence of this residual disease is associated with higher risk of relapse – a blood-based biomarker at midpoint of chemotherapy that is associated with pCR could have important utility in systemic treatment decisions.

While the focus of this study was to examine whether ctDNA and CTC surveillance early in treatment course was associated with response to neoadjuvant chemotherapy in patients with TNBC, we also aimed to evaluate the impact of ctDNA and CTC on predicting outcomes, namely recurrence-free and overall survival.

## Methods

### Study design

This was an IRB-approved study (Protocol 2014–0185) of patients with biopsy-proven triple negative (ER/PR and HER2-negative) breast cancer presenting to The University of Texas MD Anderson Cancer Center (Houston, TX, USA) from 2017 to 2021. This study was embedded within a randomized controlled trial evaluating use of a patient-specific genomic signature algorithm to predict sensitivity to standard of care versus personalized treatment regimens in patients with newly diagnosed TNBC (NCT02276443). One of the exploratory objectives focused on use of biomarkers to predict chemotherapy sensitivity. All patients received standard anthracycline-based neoadjuvant chemotherapy (NAC) for 4 cycles. Therapeutic response was assessed serially via US and/or MRI at baseline prior to therapy, after 2 cycles, and after 4 cycles of anthracycline-based NAC. Patients with ≥ 70% volumetric change in tumor by imaging were considered chemo-sensitive and proceeded to taxane ± platinum-based regimens. Those with < 70% volumetric change on imaging or developed progression of disease during treatment were considered chemo-insensitive and offered to participate in clinical trials of experimental targeted therapy based on patient-specific tumor molecular profiling. All patients underwent definitive surgical resection after completion of neoadjuvant systemic therapy. Those with stage IV disease, prior history of invasive breast cancer or other metastatic cancer within five years of enrollment, grade II or higher peripheral neuropathy, Zubrod performance status > 2, and history of serious cardiac events were deemed ineligible.

**Fig. 1:**
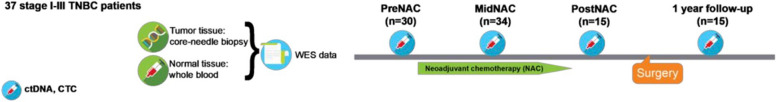
Study overview: plasma samples (n) were collected at all above timepoints to detect ctDNA and CTCs in a cohort of 37 patients with TNBC. Ultrasound (US) was performed at pre-, mid-, and post-NAC timepoints

Patients underwent prospective serial blood sample collections at four defined timepoints: pre-neoadjuvant chemotherapy (pre-NAC), 12-weeks after NAC initiation (mid-NAC, after four cycles), after NAC prior to surgery (post-NAC), and one-year after surgery (post-surgery) (Fig. 1). Response to treatment was assessed via serial ultrasound (US) imaging at pre-, mid-, and post-NAC timepoints. Dimensions of index tumors were compared to those from baseline imaging and percent reduction was calculated. Patients with more than 50% percent reduction in tumor size were considered to have at least a partial response after completion of NAC. Of note, this percentage cut-off was different from the 70% used to determine chemo-sensitivity in the overarching RCT protocol. pCR was defined as the absence of invasive tumor in the breast and axillary lymph nodes in surgical specimens. This study was conducted in accordance with principles of the Declaration of Helsinki. Informed consent was obtained from all patients prior to participation in the study. The institutional review board at The University of Texas MD Anderson Cancer Center approved this study protocol (Study #2014–0185).

### ctDNA detection

Blood samples for ctDNA were collected in 10 mL Streck Cell-Free DNA BCT® tubes with approximately 8-9 mL of peripheral venous blood. Samples were centrifuged at 2,500 rpm for 10 min at room temperature and the resulting plasma aliquoted and stored at -80 °C. ctDNA was measured from each patient’s plasma sample using a multiplex polymerase chain reaction (mPCR) ctDNA assay from Natera, Inc. (Signatera^TM^). Signatera is a tumor-informed patient-specific next-generation sequencing (NGS)-based assay that is used for the detection and quantification of ctDNA. Somatic mutations were identified from whole exome sequencing of the tumor and matched normal samples. Bespoke multiplex-PCR assays were then designed to target up to 16 single-nucleotide variants. Samples with two or more detectable variants were defined as ctDNA positive. Detected ctDNA was reported as an average variant allele fraction (VAF) and mean tumor molecules per mL of plasma (MTM/mL). Quality control was ensured through a semi-automated lab process performed by trained personnel. Samples that did not pass pre-specified quality control metrics (tumor coverage, uniformity, tumor/normal single nucleotide polymorphism genotype concordance) were excluded from further analysis.

### Isolation and enumeration of CTCs

Circulating tumor cells were quantified using CellSearch, an FDA-approved immune-based method for detecting and isolating CTCs from peripheral blood. Samples were collected using 10-mL CellSave tubes with a minimum of 7.5 mL of peripheral venous blood per patient. Briefly, peripheral blood samples undergo immune-magnetic enrichment and are positively selected for by binding to an anti-epithelial cell adhesion molecule (EpCAM)-Ab-conjugated iron nanoparticle. The cells are further stained with DAPI (4′,2-diamidino-2-phenylindole) to identify nucleated cells and a combination of CK8, CK18, and CK19 fluorescent antibodies to identify epithelial structural cytokeratin proteins. Staining with anti-CD45 allows for differentiation of circulating tumor cells from circulating white blood cells (WBCs). CTCs were then detected under direct visualization using a semi-automated fluorescent microscope.

### Statistical analyses

Wilcoxon tests and Fisher’s exact tests were used to compare differences between groups for continuous and categorical variables, respectively. Logistic regression models were used to assess for associations between ctDNA and CTC levels with pCR. Kaplan–Meier survival curves were used to predict overall survival and recurrence-free survival. Recurrence-free survival was defined as absence of development of in-situ or invasive tumors involving the breast, lymph nodes, or a distant site after definitive surgical resection. Days of overall survival and recurrence-free survival were determined from date of last follow up or date of recurrence minus date of diagnosis. Patients who remained alive (OS) or alive and recurrence-free (RFS) were censored at their last contact date. Time-to-event distributions were compared with log-rank test. All statistical analyses were performed using R version 4.2.1 with two-sided p-values considered statistically significant when < 0.05.

## Results

### Patient cohort baseline characteristics

In total, 38 patients were enrolled in the study. Of these, 1 patient was excluded due to tumor-normal tissue discordance, likely secondary to sample contamination. From the remaining 37 patients, a total of 94 serial plasma samples were collected for ctDNA analysis and a total of 101 samples for CTC enumeration. The mean age was 50 and the majority of tumors were invasive ductal carcinoma (n = 34, 91.9%, Table 1). Patients had predominantly clinical T2 disease (*n* = 25, 67.6%) and were clinically node negative at time of diagnosis (*n* = 21, 56.8%). The majority of patients had tumors with Ki-67 > 30% (*n* = 21, 56.8%). Blood samples were serially collected from patients at each timepoint: pre-NAC (*n* = 30), mid-NAC (*n* = 34), post-NAC and prior to surgery (*n* = 15), and lastly at one-year follow post-surgery (*n* = 15) (Fig. 1). Up to 40% of our cohort achieved pCR. The median length of follow-up was 44.8 months (range 12.1–64.9 months). Up to 24.3% (*n* = 9) of patients recurred and 24.3% (*n* = 9) died at last follow-up (Table 1).

**Table 1:**
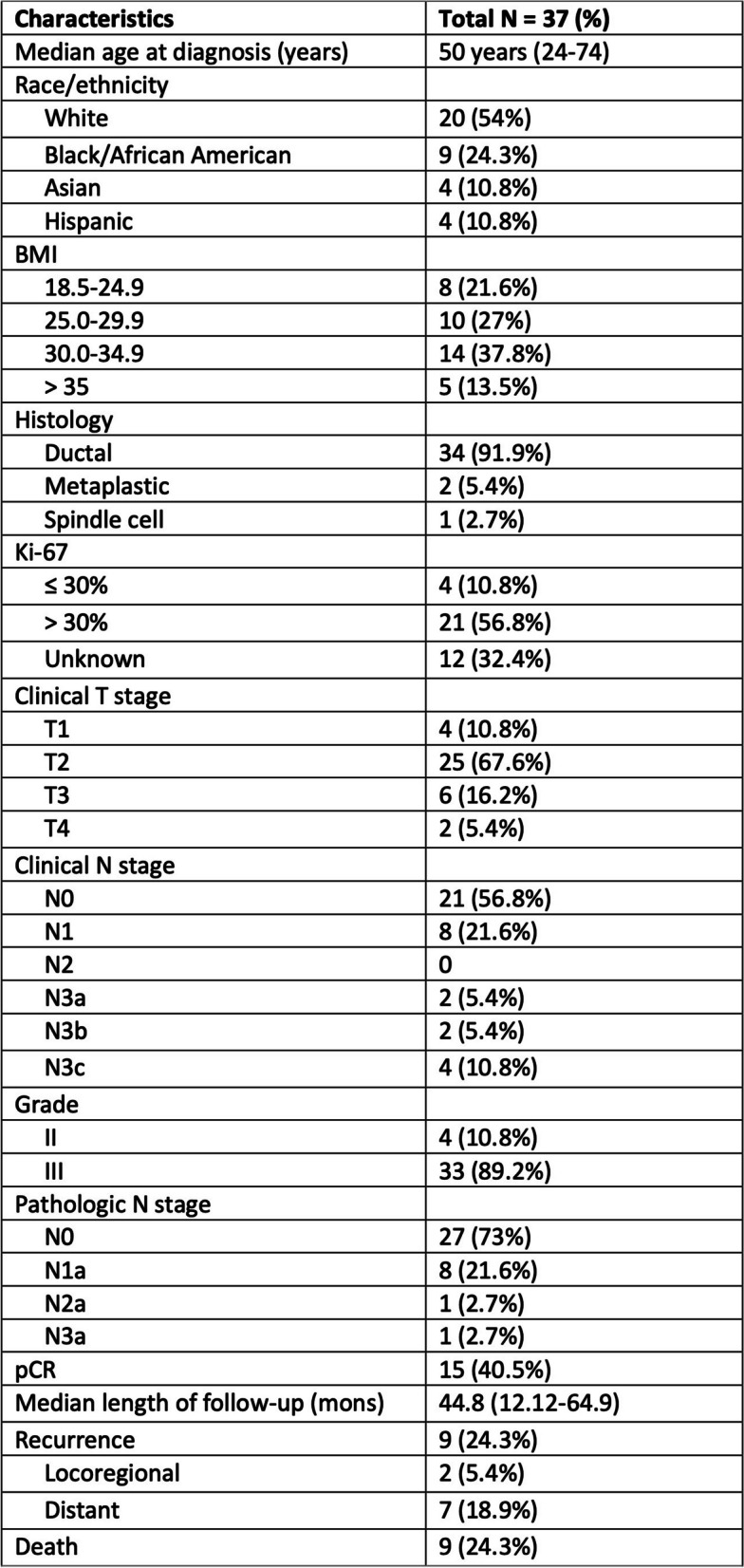
Baseline patient and clinicopathologic characteristics

### Detection of ctDNA

**Fig. 2:**
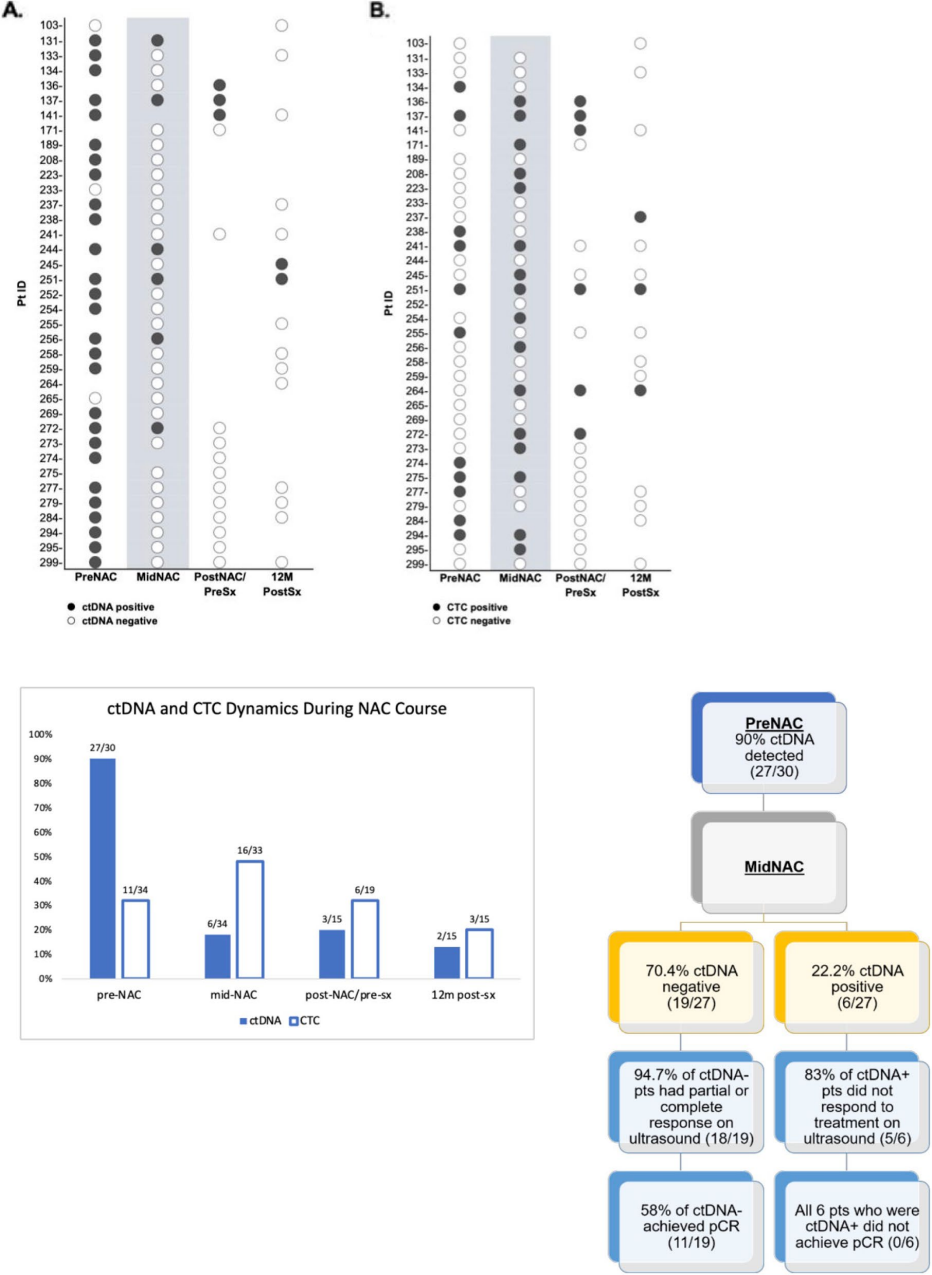
ctDNA and CTCs were quantified at pre-NAC, mid-NAC, post-NAC/pre-surgery, and 12-months post-surgery. **A**) ctDNA was detected in 90% (19/27) of patients at time of diagnosis, of whom 70.4% (19/27) achieved ctDNA clearance by mid-NAC. All 19 patients with undetectable ctDNA at the mid-NAC timepoints had PR, CR, or stable disease after completion of NAC. Importantly, 58% (11/19) of these patients achieved pCR, while none of the patients with detectable ctDNA at mid-NAC (*n*=6) achieved pCR **B**) CTCs were detected in 32.4% (11/34) of patients pre-NAC, 31.6% (16/33) mid-NAC, 31.6% (6/19) post-NAC/pre-surgery, and 20% (3/15) 12-month post-surgery. Detection of CTC was not associated with pCR. **C**) ctDNA dynamics during NAC course. **D**) ctDNA clearance by mid-NAC timepoint

The median plasma volume per patient was 4.3 mL (range: 3.0–6.0 mL) and median cfDNA concentration was 6.7 ng/mL per sample. At time of diagnosis, baseline ctDNA was detected in 90% (27/30) of patients (Fig. 2A). Among those with positive ctDNA at baseline, 70.4% (19/27) achieved ctDNA clearance by mid-NAC. Corresponding US imaging at mid-NAC demonstrated that 94.7% (18/19) of patients who achieved ctDNA clearance had evidence of partial or complete response (PR/CR). Only one case had progressive disease. Of the remaining patients who had persistent positive ctDNA at mid-NAC, only one achieved evidence of pCR by US. The presence of ctDNA persisted in 20% (3/15) of patients at post-NAC/pre-surgery timepoint and in 13.3% (2/15) at one-year follow up after surgical resection (Fig. 2A). Of these five patients, four had died at last follow-up and one developed distant lung metastasis.

### Detection of CTCs

CTCs were detected in 32.4% (11/34) of patients at the baseline (pre-NAC) timepoint (Fig. 2B). Among those with detection of CTC at baseline and available ctDNA samples, all (8/8) were also positive for ctDNA prior to treatment. Of the 11 patients with positive CTCs prior to treatment, 36.4% (4/11) achieved CTC clearance by mid-NAC. The remaining CTC dynamics throughout the treatment course are detailed in Fig. 2C.

### Association of ctDNA and CTCs with primary tumor characteristics

Presence of baseline ctDNA and/or CTCs was not significantly associated with age, BMI, race/ethnicity, tumor grade, tumor histology, clinical T and N stage, and pathologic stage on univariate analysis. Multivariate analysis was not performed due to small sample sizes.

### Association of ctDNA and CTCs with pCR

**Fig. 3:**
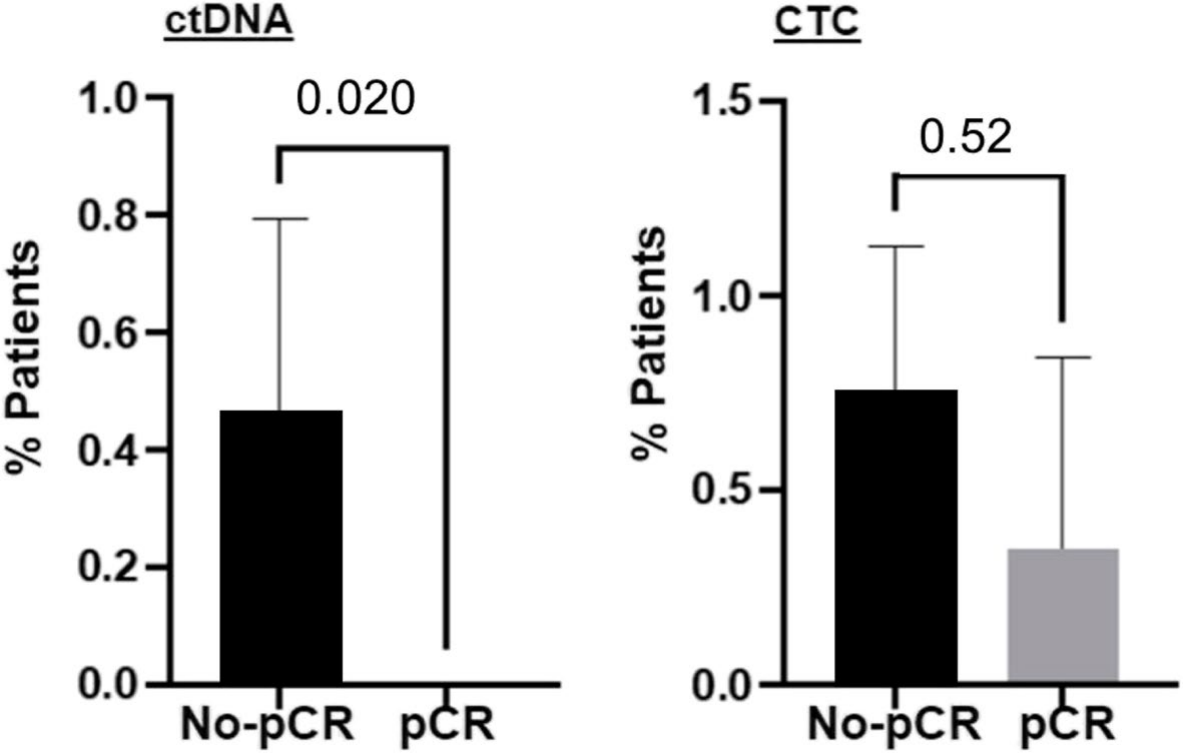
Predictive value of ctDNA and CTC status in determining pCR in TNBC patients. Analysis was limited to patients with detected ctDNA at pre-NAC and available ctDNA data at mid-NAC. Clearance of mid-treatment ctDNA was significantly associated with pCR (*p *= 0.034, left panel) while CTC status was not (*p *= 0.0825, right panel)

Of the 19 patients who achieved ctDNA clearance at mid-NAC blood draw, 58% (11/19) achieved pCR (Fig. 2D). Conversely, none of the patients with detectable ctDNA (*n* = 6) achieved pCR. The level of ctDNA and CTC at baseline and mid-treatment was not associated with achievement of pCR. However, for patients with positive ctDNA at baseline, clearance of ctDNA at mid-NAC was significantly associated with pCR (*p* = 0.02, Fig. 3). In contrast, this trend was not seen for CTC clearance at mid-treatment (*p* = 0.52, Fig. 3).

### Association of ctDNA and CTCs with tumor-volume reduction on US

There was no significant association between baseline ctDNA and/or CTC positivity and tumor volume reduction on US (*p* = 0.91 and *p* = 0.94, respectively) at completion of NAC. However, absence of ctDNA at mid-treatment was significantly associated with a higher reduction in tumor size (*p* = 0.0058). Furthermore, among patients with positive baseline ctDNA, clearance by mid-treatment was significantly associated with radiographic response by US (*p* = 0.002).

### Association of ctDNA and CTC with survival

**Fig. 4:**
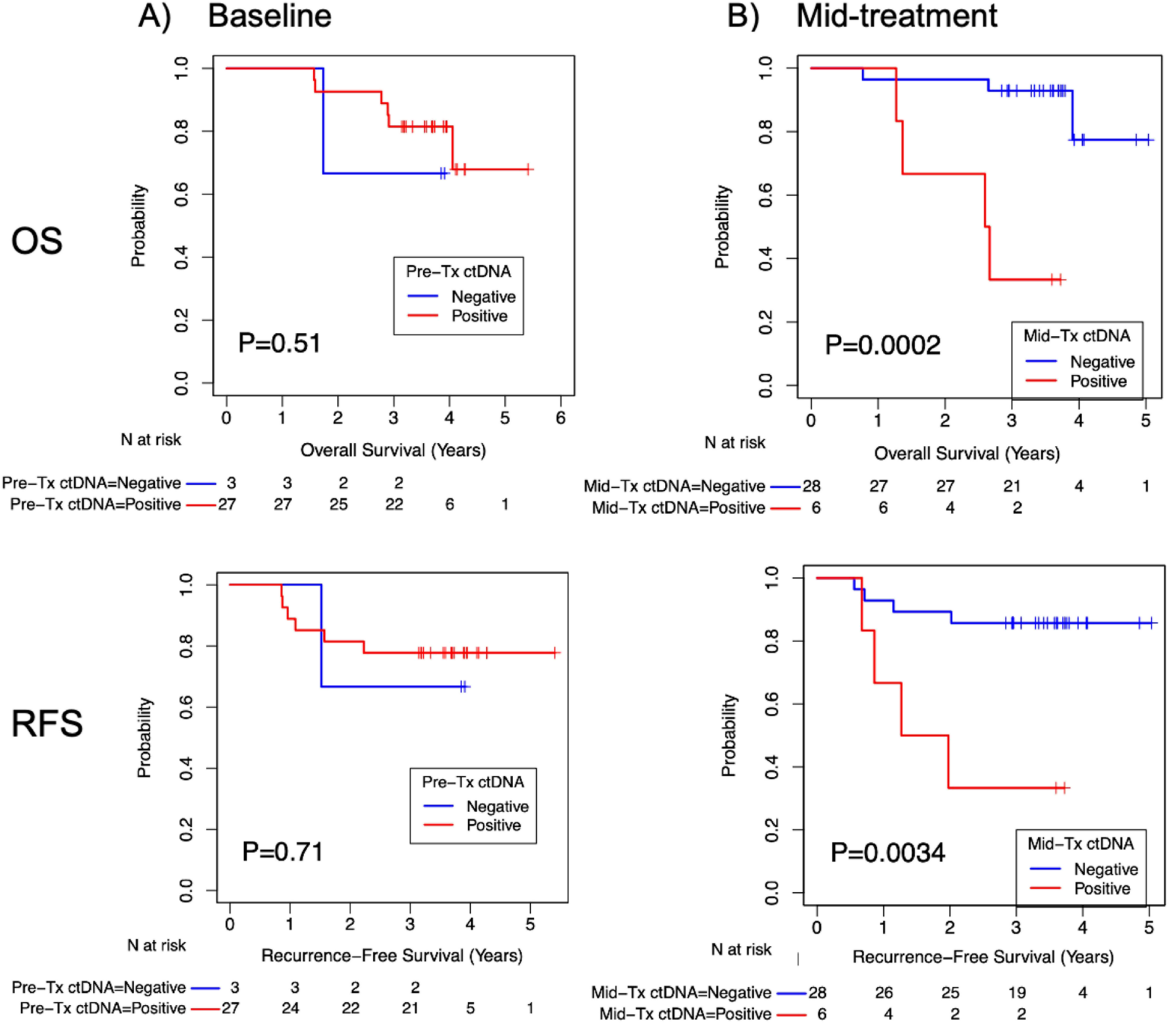
Kaplan-Meier estimates of overall and recurrence-free survival base on **A**) baseline ctDNA and **B**) mid-treatment ctdDNA. There was no significant difference in OS or PFS with ctDNA positivity at baseline. Patients with presence of mid-treatment ctDNA had significantly worse OS and RFS compared to patients with negative mid-treatment ctDNA

**Fig. 5:**
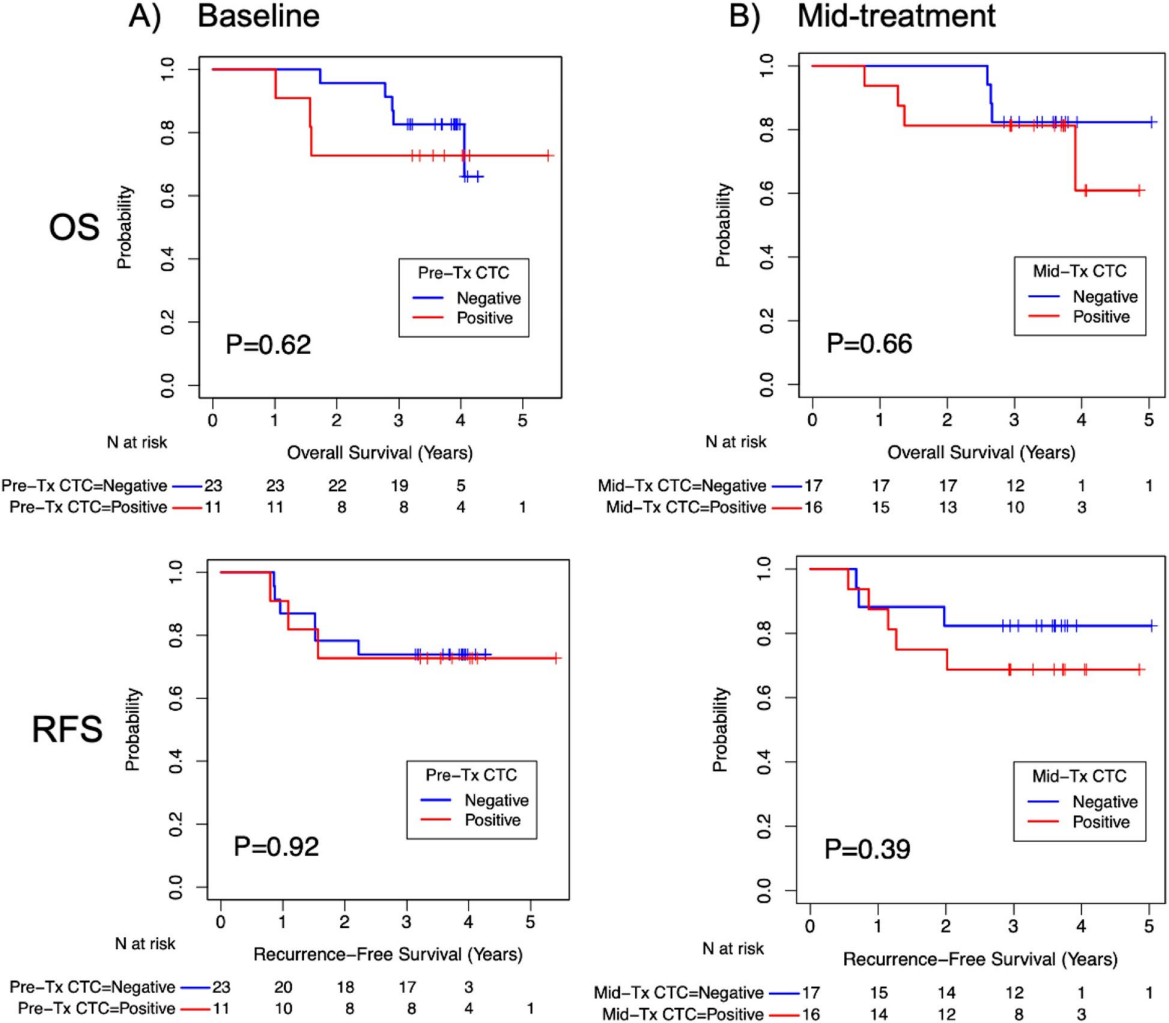
Kaplan-Meier estimates of overall and recurrence-free survival base on **A**) baseline ctDNA and **B**) mid-treatment CTC status. There was no significant difference in OS or PFS and RFS between CTC negative and positive cohorts at baseline mid-treatment

There was no difference in OS or RFS in patients with presence of ctDNA and CTC at baseline (Figs. 4 and 5). However, presence of ctDNA at mid-NAC timepoint was significantly associated with shorter OS and RFS at 5-years, compared to patients with negative ctDNA (33.3% vs. 77.4% survival at 5 years, *p* = 0.0002 and 33.3% vs. 85.7% survival at 5 years, *p* = 0.0034, respectively). No differences in OS or RFS were observed with detection of CTC at the mid-NAC timepoint (Fig. 5).

## Discussion

In this cohort of patients with TNBC, clearance of ctDNA at mid-NAC timepoint was significantly associated with pCR. Additionally, detection of ctDNA at this timepoint was associated with reduced overall survival and recurrence-free survival compared to patients with negative ctDNA. Our results demonstrate the utility of ctDNA measurement early in treatment course among patients with TNBC, an aggressive subtype with a high risk of relapse. Importantly, surveillance of ctDNA at the mid-treatment timepoint creates a window of opportunity to transition to alternative therapies in a subset of patients with poor response to traditional chemotherapy.

Our findings here are in line with current studies in the literature [10, 20, 24, 25]. Cailleux et al. collected serial plasma samples from 44 patients with early-stage breast cancer and found that detection of ctDNA at baseline prior to surgery and at last follow-up was associated with shorter event-free survival [26]. While their baseline ctDNA detection rate was considerably lower than that in our study (58% vs 90%), this discrepancy may be explained by the higher percentage of HR-positive/HER2-positive disease in their study cohort. Prior studies have demonstrated differences in ctDNA levels by receptor subtypes, with triple negative disease exhibiting relatively higher ctDNA levels and HR-positive/HER2-negative disease with lower ctDNA levels [27]. Likewise, Garcia-Murillas et al. assessed ctDNA levels in a prospective cohort of 55 patients with early breast cancer using digital droplet PCR [20]. Their results demonstrated that detection of ctDNA in serial postsurgical samples was associated with early relapse. However, they also found that baseline ctDNA detection and abundance was not associated with disease-free survival (DFS) or early relapse. In one of the largest studies focused on triple negative disease, secondary analysis of the BRE12-158 randomized clinical trial demonstrated that presence of ctDNA in 196 patients with early-stage TNBC after neoadjuvant chemotherapy and surgical resection was significantly associated with worse distant disease-free survival, DFS, and OS [28]. While the combination of ctDNA and CTC positivity was prognostic, the authors found that presence of CTCs alone was not. Similarly, in our cohort, there was no difference in OS or DFS based on CTC positivity at baseline or mid-treatment.

Collectively, studies in the literature have brought forth convincing evidence demonstrating the prognostic value of ctDNA in TNBC [20, 24, 25, 28]. However, the predictive value of liquid biopsy remains a field under active investigation [22, 23]. In our study, assessment of serial ctDNA and CTC status early in treatment course prior to surgical resection allows for evaluation of its use as a blood-based predictive biomarker. Of the 19 patients who achieved ctDNA clearance at mid-treatment, almost all had evidence of partial or complete response on US post-NAC and more than half achieved pCR. Importantly, none of the patients with detectable mid-treatment ctDNA achieved pCR (15/15, 100% specificity). However, of the 27 patients without recurrence, only three remained ctDNA positive at mid-treatment (24/27, 89% specificity). Since these patients would continue with the remaining NAC regimen, and in the event of unfavorable response at surgery would likely receive adjuvant therapy, ctDNA-positivity at mid-treatment may indicate a high probability of recurrence that could be mitigated with targeted or experimental therapies. As such, the clinical utility in mid-treatment ctDNA may lie in its ability to rule out residual disease and possible recurrence. For the 30% of patients in our study cohort who did not achieve ctDNA clearance at mid-NAC timepoint, transitioning to second-line or investigative therapies could maximize their oncologic outcomes. Importantly, these patients may be classified as poor responders, whereby serial monitoring of ctDNA provides lead time to modify treatments in the neoadjuvant setting.

It is worth noting that our results emphasize the importance of serial collection of samples throughout the disease course. Whether ctDNA and CTCs are analyzed in the neoadjuvant or adjuvant setting, a single timepoint at pre-treatment baseline or post-surgical surveillance offers limited information. The predictive value of liquid biopsy could be maximized by monitoring dynamic changes over time as patients undergo their treatment course. In a subset analysis of the I-SPY2 trial, the authors found that within the TNBC cohort, early clearance of ctDNA at three-weeks after NAC initiation was a significant predictor of pCR and residual cancer burden (RCB), compared to clearance at the 12-week mark [23]. These results suggest that monitoring ctDNA at even earlier timepoints during NAC can help identify an optimal window with the maximal amount of lead time for intervention. Furthermore, it is interesting in our study that CTCs failed to offer predictive or prognostic value in this cohort of TNBC patients. Several larger studies including breast cancer of all subtypes have previously demonstrated that presence of CTCs was an independent prognostic factor for overall survival [29, 30]. However, in this small subset of early-stage triple negative patients, the predictive and prognostic value of CTCs failed to reach significance. This finding may be due to smaller cohort size or the fact that majority of our patients had early stage T1-2 (78%) N0 disease (56.8%). Previous studies have similarly demonstrated that ctDNA might have greater sensitivity and correlation with changes in tumor burden compared to CTCs [31]. From a logistical standpoint, monitoring treatment response and disease burden may be more feasible with ctDNA as sample collection of plasma is easily attainable and does not require isolation of a specific cell population. Nevertheless, more work is needed to further characterize use of ctDNA and CTCs both alone and together as synergistic biomarkers throughout the neoadjuvant period in future prospective studies.

As with any study, there are limitations that warrant further discussion. For one, this study was conducted at a single-institution and thus has limited generalizability. However, few studies have evaluated the role of serial liquid biopsy in the neoadjuvant setting in patients with TNBC and thus, we believe our findings hold important implications for patients with this aggressive tumor subtype. Second, as this study was designed as an observational trial, we were unable to control for impact of confounders, such as variations in systemic treatment regimens, particularly those deemed chemo-insensitive and subsequently received experimental targeted therapies, on ctDNA and CTC clearance. Several randomized prospective trials currently in progress will provide more clarity on clinical utility of ctDNA in disease monitoring and tailoring of treatment regimens [32]. Third, given that our follow-up period was a median of 44 months, there may be missed future relapses beyond this time period. However, studies indicate that risk of recurrence in TNBC peaks at approximately three-years and drops precipitously thereafter [3]. Lastly, our data had occasional missing timepoints and as such, we were only able to perform analysis of patients with matched samples. Future studies with a larger cohort and more complete sample collections across timepoints will help further validate our work.

This study demonstrates that serial monitoring of ctDNA in the neoadjuvant setting might be useful as both a predictive and prognostic marker in patients with TNBC. ctDNA serves as a surrogate for residual molecular disease and provides lead time for clinicians to personalize treatment regimens based on patient response. In particular, patients who are at high risk for disease progression and/or poor responders to standard systemic therapies may benefit the most from use of liquid biopsy in the neoadjuvant setting.

## Conclusion

In patients with TNBC, this study demonstrated that early clearance of ctDNA, but not CTCs, at mid-NAC treatment was associated with a higher rate of pCR. Additionally, ctDNA positivity at mid-treatment was a significant prognostic marker for reduced OS and RFS. Personalized ctDNA monitoring during NAC is feasible and may help predict treatment response and guide individualized therapies.

## Data Availability

The associated dataset from this study is available upon request from the corresponding author.

## Abbreviations

TNBC: Triple negative breast cancer
ctDNA: Circulating tumor DNA
CTC: Circulating tumor cell
NAC: Neoadjuvant chemotherapy
pCR: Pathologic complete response
HER2: Human epidermal growth factor receptor 2
US: Ultrasound
mPCR: Multiplex polymerase chain reaction
NGS: Next-generation sequencing
VAF: Variant allele fraction
MTM: Mean tumor molecules
EpCAM: Epithelial cell adhesion molecule
DAPI: 4′,2-Diamidino-2-phenylindole
WBCs: White blood cells
OS: Overall survival
RFS: Recurrence-free survival
